# An improved cognitive model of the Iowa and Soochow Gambling Tasks with regard to model fitting performance and tests of parameter consistency

**DOI:** 10.3389/fpsyg.2015.00229

**Published:** 2015-03-12

**Authors:** Junyi Dai, Rebecca Kerestes, Daniel J. Upton, Jerome R. Busemeyer, Julie C. Stout

**Affiliations:** ^1^Decision Research Laboratory, Department of Psychological and Brain Sciences, Indiana University, Bloomington, IN, USA; ^2^Center for Adaptive Rationality, Max Planck Institute for Human DevelopmentBerlin, Germany; ^3^Department of Psychiatry, University of Pittsburgh School of MedicinePittsburgh, PA, USA; ^4^Department of Psychological Sciences, Monash UniversityClayton, VIC, Australia

**Keywords:** Iowa Gambling Task, Soochow Gambling Task, cognitive modeling, parameter consistency, opiate users

## Abstract

The Iowa Gambling Task (IGT) and the Soochow Gambling Task (SGT) are two experience-based risky decision-making tasks for examining decision-making deficits in clinical populations. Several cognitive models, including the expectancy-valence learning (EVL) model and the prospect valence learning (PVL) model, have been developed to disentangle the motivational, cognitive, and response processes underlying the explicit choices in these tasks. The purpose of the current study was to develop an improved model that can fit empirical data better than the EVL and PVL models and, in addition, produce more consistent parameter estimates across the IGT and SGT. Twenty-six opiate users (mean age 34.23; SD 8.79) and 27 control participants (mean age 35; SD 10.44) completed both tasks. Eighteen cognitive models varying in evaluation, updating, and choice rules were fit to individual data and their performances were compared to that of a statistical baseline model to find a best fitting model. The results showed that the model combining the prospect utility function treating gains and losses separately, the decay-reinforcement updating rule, and the trial-independent choice rule performed the best in both tasks. Furthermore, the winning model produced more consistent individual parameter estimates across the two tasks than any of the other models.

The Iowa Gambling Task (IGT) and the Soochow Gambling Task (SGT) are two experience-based risky decision-making tasks for examining decision-making deficits in clinical populations. Several cognitive models, including the expectancy-valence learning (EVL) model and the prospect valence learning (PVL) model, have been developed to disentangle the motivational, cognitive, and response processes underlying the explicit choices in these tasks. The purpose of the current study was to develop an improved model that can fit empirical data better than the EVL and PVL models and, in addition, produce more consistent parameter estimates across the IGT and SGT. Twenty-six opiate users (mean age 34.23; SD 8.79) and 27 control participants (mean age 35; SD 10.44) completed both tasks. Eighteen cognitive models varying in evaluation, updating, and choice rules were fit to individual data and their performances were compared to that of a statistical baseline model to find a best fitting model. The results showed that the model combining the prospect utility function treating gains and losses separately, the decay-reinforcement updating rule, and the trial-independent choice rule performed the best in both tasks. Furthermore, the winning model produced more consistent individual parameter estimates across the two tasks than any of the other models.

## Introduction

The Iowa Gambling Task (IGT; Bechara et al., 1994) and the Soochow Gambling Task (SGT; Chiu et al., 2008) are experience-based risky decision-making tasks. The IGT has been used in numerous studies to examine decision-making deficits in various clinical populations, such as people with brain damage (e.g., Bechara et al., 1994, 1999), neurodegenerative diseases (e.g., Stout et al., 2001), or drug abuse problems (Grant et al., 2000; Bechara et al., 2001; Bolla et al., 2003; Bechara and Martin, 2004; Stout et al., 2004; Gonzalez et al., 2007; Vassileva et al., 2007). The SGT was developed more recently to further distinguish influential factors for decisions using a scenario similar to the IGT (Chiu et al., 2008). While IGT studies produced ambivalent results in terms of the relevant impacts of gain-loss frequency and expected value (e.g., Dunn et al., 2006), the choice pattern of healthy participants in the SGT suggested that gain-loss frequency is more influential than expected value in determining preference in such tasks.

An important feature of the IGT and SGT is the complex interplay among motivational, cognitive, and response processes underlying the explicit choice behavior revealed in these tasks. Therefore, decision-making deficits in particular participant groups may be produced by deficiencies in different component processes. Various cognitive models have been examined to disentangle this interplay of psychological processes underlying decision task performance, and successful ones are then applied to clinical populations to identify reasons for disadvantageous choice patterns. Among them are the expectancy-valance learning model (EVL; Busemeyer and Stout, 2002) and the prospect valence learning model (PVL; Ahn et al., 2008), which have been successfully fitted to empirical data from a variety of healthy and clinical groups (Busemeyer and Stout, 2002; Stout et al., 2004; Yechiam et al., 2005; Lane et al., 2006; Fridberg et al., 2010).

In this study, we aimed to improve the cognitive models for the IGT and SGT in two major aspects. Compared with previous models, the improved model should not only provide better fits to individual data, but also demonstrate a better consistency in parameter estimates across the two tasks. The former is what we expect from a better model in general, while the latter is desired for a model which presumably captures the common decision processes underlying the two tasks.

The remainder of this article is organized as follows. First, we briefly describe and compare the IGT and SGT. Second, we describe modifications to the EVL and PVL models that might yield new improvements for quantifying the component decision processes. Third, we report a previously published behavioral study on IGT and SGT with both non-opiate user controls and clinical participants (i.e., opiate users), and compare the performances of various models in fitting individual data from the empirical study. We also report results from parameter consistency tests across tasks on the various models. The article concludes with a discussion on the implications of the new model and future research orientations.

### The IGT and SGT

The IGT was initially developed by Bechara et al. (1994) as a tool to simulate real-world risky decision-making and detect decision-making deficits of patients with brain damage. The task requires participants to choose a card from one of four decks (labeled decks A, B, C, and D respectively) on each trial and the total number of trials is unknown to participants. When a card is chosen, the payoff of that card is revealed^1^. The goal of the task is to maximize the total payoff. Some of the cards produce a pure gain (e.g., winning $0.50), while others lead to a mixture of gain and loss (e.g., winning $1 but at the same time losing $3). The cards within each deck yield the same amounts of gain but different amounts of possible loss (see **Table 1**). Specifically, each card in decks A and B yields a gain of $1 when turned over, while each card in decks C and D yields $0.50. For decks A and C, five out of each set of 10 trials produce a loss in addition to a gain. For decks B and D, only one out of each set of 10 trials produces a simultaneous loss. The amounts of potential loss are manipulated so that the expected values of decks A and B are negative (i.e., losing $2.5 in each set of 10 trials) while those of decks C and D are positive (i.e., gaining $2.5 in each set of 10 trials). The positions of trials yielding a loss within each set of 10 trials are randomized. In summary, decks C and D are better than decks A and B in terms of long-term net gain, and therefore the former are typically called the advantageous or good decks while the latter are disadvantageous or bad ones. On the other hand, decks B and D produce net gains more frequently than decks A and C.

**Table 1:** The payoff distribution of the IGT.

Deck	A	B	C	D
Expected value of 10 trials ($)	-2.50	-2.50	2.50	2.50
Gain from each trial ($)	1.00	1.00	0.50	0.50
Number of loss(es) in each set of 10 trials	5	1	5	1
Loss amount(s) in each set of 10 trials ($)	-1.50	-12.50	-0.25	-2.50
	-2.00		-0.50	
	-2.50		-0.50	
	-3.00		-0.50	
	-3.50		-0.75	

A typical finding in the initial application of the IGT to clinical populations was that healthy people tended to choose the good decks (i.e., decks C and D) more frequently than the bad ones (i.e., decks A and B) after gaining experience with the task, but participants with brain damage to the ventromedial prefrontal cortex (vmPFC) kept choosing the bad decks throughout the whole experiment. Bechara and colleagues (Damasio, 1994; Bechara et al., 1996) interpreted this pattern as a demonstration that people with damage to vmPFC cannot accumulate information from previous experience to foresee the long-term value of a specific deck and attributed this deficit to the incapability of producing a somatic marker to guide future decisions. However, Lin et al. (2007) and Chiu et al. (2008) questioned this interpretation as well as the design of IGT, arguing that there is a severe confounding between long-term outcome (i.e., expected value) and gain-loss frequency variables in the IGT (see also Dunn et al., 2006). Consequently, the preference for the good decks among healthy people may be partly caused by the fact that deck D produces a positive expected value as well as more net gains. This argument was supported by the phenomenon of “prominent deck B” (Toplak et al., 2005; Lin et al., 2007; see Dunn et al., 2006, for a review), which suggested that healthy people also tend to choose deck B more frequently than the somatic marker hypothesis predicts. As a result, Chiu et al. (2008) designed the SGT to eliminate the confounding between long-term outcome and gain-loss frequency.

The SGT has the same surface characteristics and goal as the IGT, as well as the same expected value of each deck. However, the payoff distribution of the SGT was modified to redress the confounding in the IGT. Specifically, each card in the SGT always produces either a gain or a loss (see **Table 2**). For decks A and B, four out of each set of five trials produce a gain while the remaining one produces a large amount of loss to make the expected values of these two decks negative. In contrast, for decks C and D, four out of each set of five trials produce a loss but the remaining one produces a large amount of gain so that the expected values are positive. In this way, decks with a positive expect value also produce (net) losses more frequently. Finally, the order of payoffs is randomized within each set of five instead of 10 trials.

**Table 2:** The payoff distribution of the SGT.

Deck	A	B	C	D
Expected value of 10 trials ($)	-2.50	-2.50	2.50	2.50
Payoffs in each set of five trials ($)	1.00	0.50	-1.00	-0.50
	1.00	0.50	-1.00	-0.50
	1.00	0.50	-1.00	-0.50
	1.00	0.50	-1.00	-0.50
	-5.25	-3.25	5.25	3.25

Chiu et al. (2008) found that, just like clinical participants, healthy people tended to choose the bad decks more often than the good ones in the SGT. Therefore, they argued that gain-loss frequency is more important than long-term outcome in predicting choice behavior in the IGT and SGT. This was echoed by a recent review of the IGT in healthy participants by Steingroever et al. (2013), which questioned the assumption that healthy people learn to prefer the options with positive expected values. Despite the apparent similarities between the IGT and SGT, the different payoff distributions warrant the use of both tasks in a single study to better understand risky choices from experience.

### Cognitive Models of the IGT and SGT

#### The EVL and PVL Models

Various cognitive models of the IGT and SGT have been evaluated and compared previously in terms of their descriptive accuracy for the empirical choice pattern of healthy and clinical participants. Among them, the EVL model (Busemeyer and Stout, 2002) and the PVL model (Ahn et al., 2008) appeared to be the most successful and widely used ones. Both models are built upon three general assumptions. First, participants evaluate the positive and/or negative payoffs produced by their choice on each trial with a unidimensional utility function. Second, based on the utility of experienced payoff(s) on each trial, expectation about the utility of each deck is updated with a specific learning rule. Third, the expected utility associated with each deck then serves as an input to a probabilistic function which determines the choice probability of each deck on the next trial. In other words, the explicit behavior in the IGT and SGT is determined by the interplay of three processes, i.e., the motivational process (utility evaluation), the cognitive process (expectation updating), and the response process (deck choosing).

According to the EVL model, the evaluation process is governed by the following weighted utility function

u(t)=(1−W)⋅win(t)−W⋅loss(t)⁢           (1)

in which *win(t)* and *loss(t)* represent the amounts of gain and loss on trial *t* respectively, and *W* is an attention weight parameter which denotes the weight participants place on losses as opposed to gains. It is constrained between 0 and 1; the higher it is, the more attention one puts on losses than gains. We can also interpret Equation 1 as a piecewise linear utility function with an implication that participants evaluate gains and losses separately.

The expectation updating rule involved in the EVL model is the following delta-learning rule (Rescorla and Wagner, 1972),

$$E_{j}\left(t\right)=E_{j}\left(t-1\right)+A\cdot \delta_{j}\left(t\right)\cdot \left[u\left(t\right)-E_{j}\left(t-1\right)\right]\;\;\;\;\;\;\;\;\;\;\left(2\right)$$

in which *E_j_(t)* represents the expectancy or expected utility for deck *j* on trial *t* and *A* is an updating parameter denoting the influential power of the current outcome on the expectancy for a deck. The value of *A* should be constrained between 0 and 1 so that the new expectancy after updating is bounded between the old expectancy and the utility of the immediate outcome. The variable δ_j_*(t)* in Equation 2 is a dummy variable indicating the deck chosen on trial *t*. For example, if deck C is chosen on trial t, then δ_C_*(t)* = 1, and δ_j_*(t)* = 0 for *j* = A, B, D. When *A* equals 0, the expectancy for each deck will not change (i.e., E_j_(t) = E_j_(t - 1)); when *A* equals 1, the expectancy for the chosen deck will be identical to the utility of the immediate outcome and those for the other decks will remain unchanged.

Finally, the choice rule assumed by the EVL model is a trial-dependent ratio-of-strength rule (Luce, 1959). Specifically, the choice probability of each deck on trial *t* + 1 is

$$\mathrm{Pr}[D\left(t+1\right)=j]=\frac{e^{\theta (t)\cdot E_{j}\left(t\right)}}{\Sigma_{j}e^{\theta (t)\cdot E_{j}\left(t\right)}}\;\;\;\;(3)$$

in which *D(t* + *1)* = *j* represents choosing deck *j* on trial *t* + 1 and θ*(t)* is a sensitivity parameter which determines the sensitivity of choice probabilities to expectancies on trial t. Equation 3 suggests that the higher the expectancy of a deck is, the more likely it will be chosen on the next trial. The trial-dependent choice (TDC) rule further assumes that

$$\theta \left(t\right)={\left(t/10\right)}^{c}\,\;\;\;\;\;\;\;\;\;\left(4\right)$$

Ahn et al. (2008) extended the literature by further exploring different formalizations of each of the three processes in an attempt to find a better model in terms of both descriptive and predictive accuracy for the IGT and SGT. Specifically, they tried a utility function based on the prospect theory (Kahneman and Tversky, 1979) in addition to the original piecewise linear utility function in the EVL, a decay-reinforcement learning (DRL) rule (Erev and Roth, 1998) in addition to the original delta-learning rule, and a trial-independent choice (TIC) rule as well as the original TDC rule.

According to the prospect utility function in Ahn et al. (2008),

$$u(t)=\{\begin{array}{c} {\begin{array}{c} x\left(t\right) \end{array}}^{\alpha }\,\;\;\;if\,x\left(t\right)\geq 0\\ -\lambda {\left|x\left(t\right)\right|}^{\alpha }\,\;if\,x\left(t\right)<0 \end{array}\,\;\;\;\;\;\;\;\;\;(5)$$

in which x(t) is the net payoff (i.e., *win(t)* - |*loss(t)*|) on trial *t*, α is the exponent of the power function which prescribes the shape of the utility function, and λ is a loss aversion parameter. The value of α is typically constrained between 0 and 1. When α equals 0, the prospect utility function reduces into a step function with all net gains producing the same positive utility (i.e., 1) and all net losses producing the same negative utility (i.e., –λ). When α equals 1, the utility of a net gain is equal to its objective value (i.e., *x(t)*), and the utility of a net loss is proportional to its objective value with λ serving as the proportional constant. When α is between 0 and 1 exclusively, the utility function is curved with diminishing marginal utility. The loss aversion parameter λ denotes how much an individual participant is averse to losses relative to his/her degree of preference toward gains of the same magnitude. A value of λ greater than 1 indicates that the negative utility of a loss would more than counterbalance the positive utility of a gain of the same magnitude, while a value of λ smaller than 1 suggests the opposite. The specific form of prospect utility function explored by Ahn et al. (2008) suggests that only the net payoffs are evaluated, whereas the utility function of the EVL model implies that gains and losses are evaluated separately before combined together.

The DRL rule in the PVL model suggests that in the short period between two successive trials, the expectancy resulted from the first trial decays and the updated expectancy after the second trial is a sum of the decayed expectancy and the utility of the current outcome. Specifically,

$$E_{j}\left(t\right)=A\cdot E_{j}\left(t-1\right)+\delta_{j}\left(t\right)\cdot u\left(t\right)\,\;\;\;\;\;\;\;\;\;\;\;\;\left(6\right)$$

in which *A* is a recency parameter and δ_j_*(t)* is a dummy variable as in the delta-learning rule. Unlike the delta learning rule, the DRL rule implies that the expectancies of the unchosen decks would decrease on each trial.

Finally, the TIC rule explored by Ahn et al. (2008) assumes that θ(*t*) is invariant across trials. Specifically,

$$\theta \left(t\right)=\theta =3^{c}\,-1\;\;\;\;\;\;\;\;\;\left(7\right)$$

in which *c* is a consistency parameter as in the EVL model. The higher its value is, the more consistent one’s choice will be with the expectancies of the four decks. When c equals 0, θ = 0, suggesting that choice among the four decks will be totally random no matter how different their expectancies are. When c is relative large, θ will be quite big, suggesting that people will choose the deck with the highest expectancy almost for sure. The results of Ahn et al. (2008) favored a model combining the prospect utility function, DRL rule, and TIC rule. The resultant model is usually called the PVL model^2^.

#### Alternative Cognitive Models of the IGT and SGT

Although the EVL and PVL models have been successfully applied to various populations to disentangle the interplay between component processes underlying the IGT and SGT, there are other ways to model these two tasks. Indeed, the common structure of these two models suggests using alternative utility functions, updating rules, and/or choice rules to generate potentially better models. Consequently, in this article we propose a new utility function and a new updating rule, which will be combined with complementary components in the EVL and PVL models to create new cognitive models of the IGT and SGT.

According to the prospect utility function in the PVL model, the utility of an outcome with the same amounts of gain and loss is always zero. This is due to the fact that the net outcome is zero under this condition and only net outcome is evaluated according to the PVL model. In contrast, the same property holds for the weighted utility function of the EVL model only when the attention weight parameter, W, equals 0.5 and thus gains and losses attract the same amounts of attention. When selecting a card leads to both gain and loss of the same magnitude, a participant’s overall feeling may not be neutral because, for example, the sadness associated with the loss may not be completely offset by the gain. Here, we propose an alternative form of prospect utility function that combines features of utility functions in both the EVL and PVL models,

$$u\left(t\right)={\left[win\left(t\right)\right]}^{\alpha }-\gamma {\left[\left|loss\left(t\right)\right|\right]}^{\alpha }\,\;\;\;\;\;\;\;\;\;\;\;\left(8\right)$$

in which *win(t)* and *loss(t)* represent the amounts of gain and loss on trial *t*, and α and γ have the same meanings as in the PVL model. On the one hand, like the weighted utility function, this utility function evaluates gains and losses separately before aggregating the results to generate a comprehensive utility. On the other hand, the new utility function retains the assumptions of prospect theory concerning non-linearity (i.e., α) and loss aversion (i.e., γ).

Other modifications incorporating features of both EVL and PVL models can be applied to the updating rule. According to the updating rule of the EVL model (i.e., the delta learning rule), after a card from a specific deck is turned over, the updated expectancy of the selected deck should lie between its previous expectancy and the utility of the current outcome. In contrast, the updating rule of the PVL model (i.e., the DRL rule) suggests that participants will add the utility of the current outcome to the (decayed) expectancy of the selected deck to update its expectancy. One potential problem with the DRL rule is that the updated expectancy of the selected deck can be larger in absolute magnitude than both its previous expectancy and the utility of the current outcome. In other words, the updated expectancy is not reasonably bounded. For example, suppose *A* = 0.9, *E_j_(t–1)* = 10, and *u(t)* = 5 in Equation 6 for a chosen deck. Then *E_j_(t)* = 0.9 × 10 + 5 = 14, which is larger than both *E_j_(t–1)* and *u(t)*. To get around this potential problem, explore a new learning rule that incorporates the features of both delta and DRL rules,

$$E_{j}\left(t\right)=\left(1-D\right)\cdot E_{j}\left(t-1\right)+A\cdot \delta_{j}\left(t\right)\cdot \left[u\left(t\right)-(1-D)\cdot E_{j}\left(t-1\right)\right]\,\;\;\;\;\;\;\;\;\;\;\;\;\;\;\;\;\;\left(9\right)$$

in which D is a decay parameter, A is an updating parameter, and δ*_j_(t)* is a dummy variable indicating the deck chosen on trial *t*^3^. This updating rule assumes mechanisms of both memory decay and delta-learning and thus might account for empirical data more accurately. We will hereafter call it the mixed updating rule.

In summary, with the new utility function and updating rule, we have a collection of three utility functions (i.e., the weighted utility function of the EVL model, the prospect utility function of the PVL model, and the alternative prospect utility function described above), three updating rules (i.e., the delta learning rule of the EVL model, the DRL rule of the PVL model, and the mixture updating rule), and two choice rules (i.e., the TDC rule of the EVL model and the TIC rule of the PVL model) to generate cognitive models of the two tasks. Forming all combinations, we evaluated and compared 18 cognitive models to find new models even better than the EVL and PVL models.

## Materials and Methods

### Participants

A total of 26 opiate users (mean age = 34.23 years, SD = 8.79) and 27 age and gender matched control participants (mean age = 35 years, SD = 10.44) were involved in this study (see **Table 3**). Opiate users were recruited from Turning Point Alcohol and Drug Centre, a community outpatient service located in Melbourne (Australia). Opiate users were treatment seeking, either currently abstinent or taking prescribed opiate substitution medication (methadone, buprenorphine). Participants were asked to abstain from illicit drugs and alcohol for 12 h prior to the testing session (excluding opiate substitution medication). If participants reported using alcohol or drugs less than 12 h before the test session, or had a blood alcohol level reading above 0.05 mg/kg on arrival, their test session was postponed for at least 1 day. Fourteen of the opiate users (54%) and five of the controls (19%) reported having a current mood disorder [among opiate users, two had major depressive disorder (MDD), two had an anxiety disorder, eight had MDD and an anxiety disorder, and two had a bipolar disorder; among controls, two had MDD, two had an anxiety disorder and one had MDD and an anxiety disorder]. Exclusion criteria for control participants were: use of illicit drugs in the previous 6 months, history of drug and/or alcohol dependence or abuse, blood alcohol level >0.05 mg/kg confirmed on arrival to the test session. In addition, any participants from either group who had a history of psychosis were excluded. All participants provided written informed consent, and the Monash University Human Ethics Committee approved all study procedures. See **Table 3** for more information concerning the sample^4^.

**Table 3:** Summary of demographic, mood, personality, and substance use variables.

	Controls (*n* = 27)		Drug users (*n* = 26)	
	*M* (SD)	*%*	*M* (SD)	*%*
Age	35 (10.44)		34.23 (8.79)	
Gender (male)		81.49		80.67
Est. IQ (WTAR)	35.15 (8.23)		32.42 (10.22)	
Education (years)*	14.74 (2.93)		12.25 (3.46)	
Employed*		66.67		11.54
Head inj. requiring hospital*		3.70		38.46
Mood/anxiety dis. (depression and/or anxiety)*		18.51		53.85
Anxiety (past week; HADS)*	5.41 (2.42)		9.73 (3.66)	
Depression (past week; HADS)*	3.11 (3.09)		7.50 (3.42)	
Impulsivity (Eysenck I7)*	5.41 (3.65)		10.46 (4.62)	
Antisociality (MMPI-PD)*	15.52 (4.50)		24.44 (6.31)	
**Alcohol**
* Past month use*		59.26		59.26
* Past month use (numb. days)*	3.22 (5.77)		7.42 (9.12)	
* Lifetime use (years)*	11.70 (10.78)		13.69 (8.69)	
* Problems (MAST)*	0.44 (0.66)		8.23 (6.71)	
**Tobacco**
* Never*		74.10		3.85
* Quit*		11.11		0
* Current (occasional)*		7.41		0
* Current (daily)*		7.41		96.15
**Cannabis**
* Past month use*		0		42.31
* Past month use (numb. days)*	0		8.15 (11.75)	
* Lifetime use (years)*	1.74 (5.35)		8.81 (7.84)	
**Amphetamine**
* Past month use*		0		23.10
* Past month use (numb. days)*	0		0.50 (1.14)	
* Lifetime use (years)*	0		5.04 (6.45)	
**Heroin**
* Past month use*		0		73.10
* Past month use (numb. days)*	0		5.85 (6.44)	
* Lifetime use (years)*	0		9.35 (6.75)	
**Prescr. Methadone (current)**
* Past month use*		0		46.15
* Past month use (numb. days)*	0		13.69 (15)	
* Lifetime use (years)*	0		1.96 (3.23)	
Parent hist. (sub. problems)*		3.70		50
Illicit drug problems (DAST)	0.30 (0.61)		14.54 (4.34)	

*p 0.05.

### Procedure

The participants completed computerized versions of the IGT and SGT. The order of tasks was counterbalanced across participants. Each task had 120 trials with an unlimited number of cards in each deck. Participants were given a starting balance of $20.00 and received any money earned above this balance at the end of the task. They could not lose any money. The total balance was updated on-screen after every selection and participants were also provided with feedback about the net change in balance after every 20 trials. Each trial was participant-initiated, and there were no time restrictions. Decks were positioned on the computer screen, from left to right, randomly across participants.

### Model Comparison Analyses

#### Maximum Likelihood Estimation

A total of 18 cognitive models (3 utility functions × 3 updating rules × 2 choice rules) were fit to the choice data of each individual in each task. We used these models to predict choice probabilities of the four decks on each trial given the outcomes experienced on previous trials. The one-step-ahead predictions were then employed to evaluate the performance of each model. Specifically, we defined the likelihood of the observed choice sequence of each participant as the product of the predicted choice probabilities of the decks actually chosen across trials^5^ and we used maximum-likelihood estimation to find the best parameter values for each model. The log likelihood of the observed sequence is defined as

$${LL}_{M}=\overset{n-1}{\underset{t=1}{\Sigma }}\underset{j}{\Sigma }\;\ln\left(\mathrm{Pr}\left[D_{j}\left(t+1\right)\right]\right)\cdot \delta_{j}\left(t+1\right)\,\;\;\;\;\;\;\;\;\left(10\right)$$

In the above equation, *n* denotes the number of trials, Pr[*D_j_*(*t* + 1)] represents the predicted choice probability of deck *j* on trial *t* + 1 given the sequence of choices and outcomes up to and including trial *t*, δ_j_(*t* + 1) is a dummy variable with a value of 1 if deck *j* is chosen on trial *t* + 1 and 0 otherwise, and the second summation is across the four decks. A combination of grid-search with 50 different starting positions and simplex search method (Nelder and Mead, 1965) was utilized to find the best parameter values.

#### Model Comparisons Using the Bayesian Information Criterion

Since models explored in this study differed in number of parameters, the Bayesian information criterion (BIC; Schwartz, 1978) was used as the main performance index for model comparison, because it considers both descriptive accuracy and model complexity. We also explored a statistical baseline model as in Busemeyer and Stout (2002) and Ahn et al. (2008). This model assumes independent choices with constant probabilities across trials and served as the reference point in our model comparison. Three free parameters are involved in this model, representing the choice probabilities of the first three decks on each trial. By definition, the choice probability of the last deck equals one minus the sum of those of the first three decks. This model suggests that people choose among the four decks without considering previous choices and outcomes and thus the choice probability of each deck remains the same throughout the whole task. Consequently, a cognitive model performs better than the baseline model only if it can account for the dependency of choices on previous choices and outcomes. The BIC difference score of a specific model compared with the baseline model^6^ is

$$BIC=2\left({\overset{\wedge }{LL}}_{M}-{\overset{\wedge }{LL}}_{Baseline}\right)-k\cdot \ln\left(n\right)\,\;\;\;\;\;\;\;\left(11\right)$$

in which 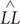 denotes the maximum log-likelihood produced by a model, *k* denotes the difference in the number of parameters and *n* is the number of data points used in fitting the models. Positive values of the BIC difference score indicate that a cognitive model outperforms the baseline model and the higher the better. A more complex model tends to produce a higher maximum log-likelihood but is also associated with a larger value of *k*. Therefore, models with more parameters do not necessarily lead to higher BIC scores.

#### Parameter Consistency Test

The implicit assumption underlying our effort to fit data from both tasks with the same model is that participants’ choices in these similar tasks are at least partly governed by the same mechanisms. Consequently, model parameters estimated from the two tasks should be positively associated since they are supposed to measure relatively stable psychological characteristics of the same individual across tasks (Yechiam and Busemeyer, 2008). To test this hypothesis, we conducted a correlational analysis between the two tasks for every parameter of each model. A good model in this respect should produce positive correlation coefficient for each parameter involved.

## Results

### Model Comparison

By comparing the various models (see **Table 4**), we obtained five key findings. First, in all cases cognitive models performed better on average than the baseline statistical model when making one-step-ahead predictions. This is not unexpected given that the baseline model assumes independent choices across trials, which seems unlikely since these tasks promote learning from feedback throughout. Evidence that the cognitive models performed better than the baseline model came from the positive mean BIC difference score of each cognitive model across both the IGT and SGT. Second, most of the cognitive models fit IGT data better than SGT data. This was true no matter whether mean BIC difference score, median BIC difference score, or percentage of positive BIC difference scores was used as a criterion. Third, in both tasks the delta learning rule was always inferior to the DRL rule and the new mixed learning rule no matter what utility function and choice rule were involved.

**Table 4:** Summary of Bayesian information criterion (BIC) difference scores of the 18 cognitive models relative to the baseline statistical model in the IGT and SGT.

Model				Task							
				IGT				SGT			
Utility function	Updating rule	Choice rule	# Parameters	M	Mdn	SD	% (BIC>0)	M	Mdn	SD	% (BIC>0)
EU	DEL	TDC	3	0.76	-1.78	24.7	45	4.40	-2.30	28.2	42
		TIC	3	2.97	-1.48	26.0	38	4.03	-2.25	28.2	36
	DRL	TDC	3	29.63	16.51	48.8	66	11.95	1.49	39.4	55
		TIC	3	33.09	18.28	50.9	64	16.87	3.52	36.9	60
	ML	TDC	4	25.07	11.46	46.6	64	11.37	1.48	35.1	51
		TIC	4	29.35	13.41	50.6	62	13.05	-1.08	36.9	49
PU	DEL	TDC	4	11.29	1.38	34.8	55	7.22	0.13	29.0	53
		TIC	4	13.72	3.11	36.4	58	7.94	1.26	27.9	53
	DRL	TDC	4	27.89	14.93	50.3	66	15.59	6.16	38.1	64
		TIC	4	31.71	16.81	49.2	66	20.82	9.97	36.5	72
	ML	TDC	5	24.93	11.93	43.2	66	15.03	7.06	34.1	60
		TIC	5	29.96	13.97	47.3	68	17.16	5.60	36.1	60
PU2	DEL	TDC	4	13.69	2.78	33.2	64	7.22	0.13	29.0	53
		TIC	4	14.68	3.97	34.4	64	7.94	1.26	27.9	53
	DRL	TDC	4	31.52	16.88	53.1	68	15.59	6.16	38.1	64
		TIC	4	35.69	15.35	53.4	74	20.82	9.97	36.5	72
	ML	TDC	5	31.14	15.22	47.2	68	15.03	7.06	34.1	60
		TIC	5	33.81	17.11	51.7	68	17.16	5.60	36.1	60

*EU, expectancy utility function; PU, prospect utility function; PU2, alternative prospect utility function; DEL, delta learning rule; DRL, decay-reinforcement learning rule; ML, mixed learning rule; TDC, trial-dependent choice rule; TIC, trial-independent choice rule; IGT, Iowa Gambling Task; SGT, Soochow Gambling Task; M, mean; Mdn, median; SD, standard deviation*.

Furthermore, when combined with the same updating and choice rules, the new utility function based on prospect theory performed better than the previous utility functions used with the EVL and PVL models. In the IGT, regardless of what updating and choice rules were utilized, the alternative prospect utility function always produced a higher average BIC difference score than the other two utility functions. In the SGT, the BIC difference scores generated from the two prospect utility functions were always identical because the task involves only a single outcome on each trial. On the other hand, the prospect utility functions were better than the piecewise weighted utility function in the EVL model in terms of average BIC difference score no matter what updating and choice rules were in force. Similar patterns could be found when median BIC difference score or percentage of positive BIC difference scores was used as the criterion. In general, the new prospect utility function performed better than the other two utility functions.

Finally, with the alternative prospect utility function, the model with DRL rule (DRL) and TIC rule appeared to be the best: it produced a higher average BIC difference score and more positive BIC difference scores than any of the other five models in both tasks. It was only inferior to the model with DRL rule and TDC rule, and the model with mixed learning rule and TIC rules, when median BIC difference score was used as the criterion and IGT data were fit. To choose among these three competing models, we further conducted pairwise comparisons in terms of the number of participants whose BIC difference scores were higher on one model than another (Broomell et al., 2011). The model with DRL and TIC again worked better in this comparison. Specifically, when comparing the DRL+TIC model with the DRL+TDC model, the former produced higher BIC difference scores on 34 participants in the IGT, whereas the latter produced higher BIC scores on only 19 participants. The corresponding numbers in the SGT were 42 and 11 respectively. Similarly, when comparing the DRL+TIC model with the model assuming mixed updating rule and TIC rule, the former produced higher BIC difference scores on 42 participants in the IGT and 49 participants in the SGT. Given the general advantage of the new model with the alternative prospect utility function, DRL rule, and TIC rule, we will hereafter treat it as the winning model and call it the PVL2 model. This model has four parameters, with higher values indicating more rapid memory decay, more consistent choices with regard to deck expectancies, higher levels of sensitivity to outcome differences, and more loss aversion respectively. Note that almost the same results from model comparison occurred when participants were divided into separate groups of opiate users and healthy controls (see Tables **Table 5** and **Table 6**).

**Table 5:** Summary of BIC difference scores of the 18 cognitive models relative to the baseline statistical model in the IGT and SGT among controls.

Model				Task							
			IGT				SGT			
Utility function	Updating rule	Choice rule	M	Mdn	SD	% (BIC>0)	M	Mdn	SD	% (BIC>0)
EU	DEL	TDC	3.14	3.82	30.9	63	8.43	-2.30	32.3	41
		TIC	6.20	0.57	33.0	52	8.44	-2.44	35.2	37
	DRL	TDC	45.30	41.11	56.4	85	18.98	10.70	45.9	63
		TIC	49.79	37.45	59.3	85	23.01	9.95	43.3	59
	ML	TDC	40.82	35.34	53.3	89	18.28	3.87	39.8	59
		TIC	45.96	32.66	58.6	81	19.12	5.15	43.9	56
PU	DEL	TDC	15.97	8.56	42.5	67	12.91	3.27	32.2	56
		TIC	19.03	12.19	45.2	70	13.73	3.19	33.9	63
	DRL	TDC	43.89	39.56	56.4	78	23.84	10.47	44.1	74
		TIC	47.87	42.63	55.6	81	29.02	16.07	41.5	78
	ML	TDC	39.49	33.36	48.4	89	23.24	13.00	37.3	70
		TIC	45.71	38.22	53.6	81	25.12	11.29	41.6	70
PU2	DEL	TDC	18.97	11.25	40.6	74	12.91	3.27	32.2	56
		TIC	21.56	12.98	42.6	74	13.73	3.19	33.9	63
	DRL	TDC	49.17	43.75	59.8	81	23.84	10.47	44.1	74
		TIC	53.41	43.49	61.7	85	29.02	16.07	41.5	78
	ML	TDC	48.03	38.12	53.0	85	23.24	13.00	37.3	70
		TIC	51.99	40.80	59.3	81	25.12	11.29	41.6	70

*EU, expectancy utility function; PU, prospect utility function; PU2, alternative prospect utility function; DEL, delta learning rule; DRL, decay-reinforcement learning rule; ML, mixed learning rule; TDC, trial-dependent choice rule; TIC, trial-independent choice rule; IGT, Iowa Gambling Task; SGT, Soochow Gambling Task; M, mean; Mdn, median; SD, standard deviation*.

**Table 6:** Summary of BIC difference scores of the 18 cognitive models relative to the baseline statistical model in the IGT and SGT among opiate users.

Model				Task							
			IGT				SGT			
Utility function	Updating rule	Choice rule	M	Mdn	SD	% (BIC>0)	M	Mdn	SD	% (BIC>0)
EU	DEL	TDC	-0.96	-4.90	16.7	29	0.67	-1.27	24.1	46
		TIC	-0.39	-4.26	15.9	23	-0.55	-2.09	18.1	35
	DRL	TDC	13.36	-1.07	33.2	46	4.65	-2.68	30.4	46
		TIC	15.75	-1.63	33.5	42	10.79	2.92	28.3	62
	ML	TDC	8.71	-6.92	32.1	38	4.19	-3.17	28.5	42
		TIC	12.10	-5.88	33.9	42	6.73	-1.46	27.4	42
PU	DEL	TDC	6.43	-0.81	24.3	42	1.31	-0.84	24.5	50
		TIC	8.21	-0.39	23.9	46	1.92	-1.21	18.7	42
	DRL	TDC	11.28	2.29	37.3	54	7.01	1.20	29.2	54
		TIC	14.92	-0.17	35.2	50	12.31	4.27	28.9	65
	ML	TDC	9.81	-3.92	31.3	42	6.51	-0.64	28.7	50
		TIC	13.60	2.58	33.5	54	8.89	0.20	27.7	50
PU2	DEL	TDC	8.21	0.97	22.9	54	1.31	-0.84	24.5	50
		TIC	7.54	1.07	21.7	54	1.92	-1.21	18.7	42
	DRL	TDC	13.18	4.75	38.3	54	7.01	1.20	29.2	54
		TIC	17.28	3.83	35.9	62	12.31	4.27	28.9	65
	ML	TDC	13.60	0.06	32.9	50	6.51	-0.64	28.7	50
		TIC	14.94	1.81	34.5	54	8.89	0.20	27.7	50

*EU, expectancy utility function; PU, prospect utility function; PU2, alternative prospect utility function; DEL, delta learning rule; DRL, decay-reinforcement learning rule; ML, mixed learning rule; TDC, trial-dependent choice rule; TIC, trial-independent choice rule; IGT, Iowa Gambling Task; SGT, Soochow Gambling Task; M, mean; Mdn, median; SD, standard deviation*.

### Parameter Consistency

**Table 7** shows the correlation coefficient for each parameter of the PVL2 model between individual estimates from the two tasks. We used Spearman’s rho coefficient since both Kolmogorov–Smirnov test and Shapiro–Wilk test of normality led to significant results on each parameter in both tasks (all *p*s < 0.05), and one-tailed tests because, according to the hypothesis on parameter consistency, we expected positive coefficients. As expected, there was a positive association between the estimates from the two tasks for each parameter of the winning model. The only other model that also produced significant correlations on all parameters was the model with expectancy utility function (i.e., the weighted utility function in the EVL model), DRL rule, and TIC rule. However, the strength of association produced by this model was lower than that of the PVL2 model (see **Table 8**)^7^. In summary, the PVL2 model outperformed all the other models with regard to parameter consistency across the IGT and SGT. Furthermore, the same pattern of associations occurred when participants were divided into groups of opiate users and healthy controls, although certain correlation coefficients might not be statistically significant due to the small sample sizes.

**Table 7:** Correlations for parameters of the PVL2 model estimated from the IGT and SGT.

Parameter	Spearman’s rho	one-tailed *p*-value	ρ^2^
Memory decay (D)	0.292	0.017	0.085
Choice consistency (c)	0.465	0.001	0.216
Outcome sensitivity (α)	0.266	0.027	0.071
Loss aversion (γ)	0.468	0.001	0.219

**Table 8:** Correlations for parameters estimated from the IGT and SGT of the model with expectancy utility function, decay-reinforcement learning rule, and trial-independent choice rule.

Parameter	Spearman’s rho	one-tailed *p*-value	ρ^2^
Memory decay (D)	0.265	0.028	0.070
Choice consistency (c)	0.355	0.005	0.126
Loss weight (W)	0.302	0.014	0.091

## Discussion

In this article, we made a systematic comparison of various models for the IGT and SGT, including the EVL and PVL models which have been widely adopted in the literature. Specifically, with the alternative prospect utility function and mixed updating rule, we generated 18 cognitive models of the IGT and SGT by factorially combining different utility functions, updating rules, and choice rules. The winning model, i.e., the PVL2 model, is similar to the PVL model but with a different implementation of the utility function of prospect theory. The BIC scores suggested that, for both healthy controls and opiate users, the PVL2 model outperformed the EVL model in both tasks and the PVL model in the IGT. These results implied that the alternative prospect utility function might provide a better approximation to the actual evaluation process than the other two utility functions. In other words, people evaluate positive and negative outcomes separately before combining the results into a comprehensive measure (as in the EVL model) and they become less sensitive to outcome difference when the absolute magnitudes of outcomes increase (as in the PVL model).

Although on average all the cognitive models performed better than the baseline model in both tasks, most of the cognitive models fit IGT data better than SGT data. Close scrutiny of the differences in payoff distribution between the two tasks revealed that the SGT not only couples low-frequency losses with negative expected values but also introduces more subtle structural properties that might induce participants to respond differently in the SGT than in the IGT. For example, it might be actually desirable in the SGT to choose the bad decks one more time after they produce a negative outcome because it is very unlikely that the next outcome will be a loss again. This is not true in the IGT, at least for deck A which produces a net loss half of the time. Similarly, people might avoid choosing the same good decks in the SGT when they just yield a positive outcome because there is a high probability that the same deck will produce a loss on the next trial. Such a tendency is clearly inconsistent with the current class of models which assume implicitly that a positive outcome would increase the choice probability of the selected deck and vice versa. This might be the major reason for the poorer performance of the models on SGT data. Future research is necessary to develop more sophisticated models incorporating this tendency.

Given that the DRL rule might produce an unbounded updated expectancy, one may wonder why this updating rule is still selected by the model comparison. Two possible explanations exist for the current result. First, although the DRL rule might produce an unbounded expectancy, this is not always true. The presumably undesirable situation occurs only when E_j_(t–1) and u(t) have the same sign and the former is no larger than the latter in absolute magnitude, or when the two terms have the same sign, the former is larger than the latter in absolute magnitude, and the decay parameter is relatively small. More important, according to the winning model, the choice probabilities of the four decks are related to the relative magnitudes of deck expectancies rather than the absolute magnitudes. Therefore, models allowing for unbounded expectancies across the four decks might lead to the same predictions on choice probabilities as those only producing bounded expectancies.

After establishing the PVL2 model as the best model with regard to model fitting performance, we examined the issue of parameter consistency for each model. Within-subject data on both tasks made it possible to investigate whether parameter estimates from the two tasks were associated with each other. The results indicated that individual estimates of each parameter in the PVL2 model were positively associated across the two tasks. This suggested that choice responses in these two tasks were at least partly governed by the same mechanisms reflected by the PVL2 model. Although a similar model with the same updating and choice rules but a different utility function (i.e., the weighted utility function) also led to significant correlation coefficient on each parameter involved, the strength of association between its parameter estimates was lower than that of the PVL2 model. Therefore, the PVL2 model still outperformed all the other models in terms of parameter consistency.

One natural question arises from the results advocating the new model: how does the model account for the differences in behavioral data between opiate users and healthy controls? For example, does the winning model suggest that the differences in behavioral data are the consequence of differential degrees of choice variability, outcome sensitivity and/or loss aversion between the two groups? For any cognitive model of the IGT and SGT, this is no doubt a critical issue to address. However, it seems premature to answer the question right now for the following reasons: (1) the complex pattern of abnormality in the current samples, and (2) the relatively small sample sizes. Future studies with larger and more homogeneous samples of opiate users and controls are necessary to provide a convincing answer to this question.

Besides modeling the IGT and SGT from a reinforcement learning perspective, previous research has also investigated the role of perseveration in these tasks (Worthy and Maddox, 2012; Worthy et al., 2013a). Recently, Worthy et al. (2013b) further explored the benefit of combining reinforcement learning with perseveration in describing observed data from the IGT. It turned out that a model with the delta learning rule and a separate term for perseveration outperformed the PVL model with the DRL rule. Furthermore, it was proposed that the DRL rule may perform better than the delta learning rule not because memory decay plays a critical role in the tasks but because the former accounts for participants’ tendency to perseverate but not the latter. Whether the same will occur to the current modeling attempt is an open question. On the one hand, with the alternative prospect utility function, treating perseveration separately may again improve the fitting performance of a model with the delta learning rule relative to a model with the decay-reinforcement rule, at least for the IGT data. On the other hand, the resultant more complicated models may perform poorly in the consistency test due to the extra parameters and more subtle interactions among all the parameters. Future studies should test models with the alternative prospect utility function and a separate term for perseveration across the IGT and SGT to advance our understanding of the mechanisms underlying these tasks.

In conclusion, our analyses on the empirical data from both healthy and clinical participants suggested that the PVL2 model with the alternative prospect utility function, DRL rule, and TIC rule performed even better than the previous best model, i.e., the PVL model, in describing individual data. In addition, the PVL2 model also produced more consistent parameter estimates across the IGT and SGT than various other models examined in this study. Consequently, we recommend the PVL2 model as a candidate model of both the IGT and SGT in future studies on these two tasks.

## Footnotes

1 The payoffs of the IGT used in this study were 1/100th of the hypothetical payoffs in the original design of Bechara et al. (1994). In this way, the participants could be paid what they actually encountered in the study. The same is true for the payoffs of the SGT used in this study.

2 The definitive feature of the PVL model is the use of the specific form of prospect utility function explored in Ahn et al. (2008). See Fridberg et al. (2010) for more information.

3 Note that we replace the recency parameter *A* in Equation 6 [i.e., Equation 4 in Ahn et al. (2008)] with (1 – *D*) and reserve symbol *A* for the updating parameter to formulize the mixed updating rule. This leads to clearer symbol system in this article and higher values of *D*, the decay parameter, actually indicate more rapid memory decay. The same is true for our formulation of the decay-reinforcement learning rule.

4 Behavioral data from this sample were reported and analyzed in Upton et al. (2012). In the current study we focus on the performance of various models with regard to the data.

5 The first trial was actually skipped in calculating likelihood of the choice sequence since all models predict equal choice probabilities (i.e., 0.25) across decks for the first trial.

6 More precisely, Equation 11 represents the change in BIC value between the cognitive model and the baseline model.

7 See Appendix for a table with the correlation coefficients for parameter estimates from the IGT and SGT for all of the models.

## Appendix

**Table A1:** Correlations for parameters estimated from the IGT and SGT for all of the models.

Model				Parameters					
Utility function	Updating rule	Choice rule	Memory decay (D)	Updating (A)	Choice consistency (c)	Outcome sensitivity (α)	Loss aversion (γ)	Loss weight (W)
EU	DEL	TDC	–	0.082	0.258ˆ*	–	–	0.150
		TIC	–	0.188	0.099	–	–	0.158
	DRL	TDC	-0.019	–	0.306ˆ*	–	–	0.197
		TIC	0.265ˆ*	–	0.355ˆ**	–	–	0.302ˆ*
	ML	TDC	0.047	-0.009	0.454ˆ**	–	–	0.260ˆ*
		TIC	0.266ˆ*	0.034	0.012	–	–	0.302ˆ*
PU	DEL	TDC	–	0.283ˆ*	0.222	0.019	0.211	–
		TIC	–	0.335ˆ**	0.174	0.145	-0.045	–
	DRL	TDC	0.298ˆ*	–	0.466ˆ**	0.110	0.261ˆ*	–
		TIC	0.337ˆ**	–	0.515ˆ**	-0.194	0.363ˆ**	–
	ML	TDC	0.209	0.252ˆ*	0.542ˆ**	0.174	0.215	–
		TIC	0.342ˆ**	0.142	0.189	-0.122	0.151	–
PU2	DEL	TDC	–	0.273ˆ*	0.178	-0.014	0.144	–
		TIC	–	0.203	0.211	0.083	0.090	–
	DRL	TDC	0.230ˆ*	–	0.466ˆ**	0.181	0.358ˆ**	–
		TIC	0.292ˆ*	–	0.465ˆ**	0.266ˆ*	0.468ˆ**	–
	ML	TDC	0.156	0.092	0.378ˆ**	0.141	0.365ˆ**	–
		TIC	0.317ˆ*	0.195	0.242ˆ*	0.192	0.211	–

*EU, expectancy utility function; PU, prospect utility function; PU2, alternative prospect utility function; DEL, delta learning rule; DRL, decay-reinforcement learning rule; ML, mixed learning rule; TDC, trial-dependent choice rule; TIC, trial-independent choice rule*.

***p* 0.05; ***p* 0.01*.

## References

[B1] Ahn W.-Y. Busemeyer J. R. Wagenmakers E.-J. Stout J. C. (2008). Comparison of decision learning models using the generalization criterion method. *Cogn. Sci.* 32 1376–1402 10.1080/03640210802352992 21585458

[B2] Bechara A. Damasio H. Tranel D. Anderson S. (1994). Insensitivity to future consequences following damage to human prefrontal cortex. *Cognition* 50 7–15 10.1016/0010-0277(94)90018-3 8039375

[B3] Bechara A. Dolan S. Denburg N. Hindes A. Anderson S. W. Nathan P. E. (2001). Decision-making deficits, linked to a dysfunctional ventromedial prefrontal cortex, revealed in alcohol and stimulant abusers. *Neuropsychologia* 9 376–389 10.1016/S0028-3932(00)00136-6 11164876

[B4] Bechara A. Martin E. M. (2004). Impaired decision making related to working memory deficits in individuals with substance addictions. *Neuropsychology* 18 152–162 10.1037/0894-4105.18.1.152 14744198

[B5] Bechara A. Tranel D. Damasio H. Damasio A. R. (1996). Failure to respond autonomically to anticipated future outcomes following damage to frontal cortex. *Cereb. Cortex* 6 215–225 10.1093/cercor/6.2.215 8670652

[B6] Bechara A. Tranel D. Hindes A. (1999). Roles of the amygdala, pre-frontal cortex, and right somatosensory/insular cortex in decision making and emotional processing. *J. Neuropsychiatry Clin. Neurosci.* 11:157.

[B7] Bolla K. I. Eldreth D. A. London E. D. Kiehl K. A. Mouratidis M. Contoreggi C. (2003). Orbitofrontal cortex dysfunction in abstinent cocaine abusers performing a decision-making task. *Neuroimage* 19 1085–1094 10.1016/S1053-8119(03)00113-7 12880834 PMC2767245

[B8] Broomell S. B. Budescu D. V. Por H.-H. (2011). Pair-wise comparisons of multiple models. *Judgm. Decis. Mak.* 6 821–831.

[B9] Busemeyer J. R. Stout J. C. (2002). A contribution of cognitive decision models to clinical assessment: decomposing performance on the bechara gambling task. *Psychol. Assess.* 14 253–262 10.1037/1040-3590.14.3.253 12214432

[B10] Chiu Y.-C. Lin C.-H. Huang J.-T. Lin S. Lee P.-L. Hsieh J.-C. (2008). Immediate gain is long-term loss: are there foresighted decision makers in the Iowa Gambling Task? *Behav. Brain Funct.* 4 13 10.1186/1744-9081-4-13 PMC2324107 18353176

[B11] Damasio A. R. (1994). *Descartes’ Error: Emotion, Reason, and the Human Brain*. New York: Putnam.

[B12] Dunn B. D. Dalgleish T. Lawrence A. D. (2006). The somatic marker hypothesis: a critical evaluation. *Neurosci. Biobehav. Rev.* 30 239–271 10.1016/j.neubiorev.2005.07.001 16197997

[B13] Erev I. Roth A. (1998). Predicting how people play games: Reinforcement learning in experimental games with unique, mixed strategy equilibria. *Am. Econ. Rev.* 88 848–881.

[B14] Fridberg D. J. Queller S. Ahn W.-Y. Kim W. Bishara A. J. Busemeyer J. R. (2010). Cognitive mechanisms underlying risky decision-making in chronic cannabis users. *J. Math. Psychol.* 54 28–38 10.1016/j.jmp.2009.10.002 20419064 PMC2858342

[B15] Gonzalez R. Bechara A. Martin E. M. (2007). Executive functions in individuals with methamphetamine or alcohol as drugs of choice: preliminary observations. *J. Clin. Exp. Neuropsychol.* 29 155–159 10.1080/13803390600582446 17365250

[B16] Grant S. Contoreggi C. London E. D. (2000). Drug abusers show impaired performance in a laboratory test of decision making. *Neuropsychologia* 38 1180–1187 10.1016/S0028-3932(99)00158-X 10838152

[B17] Kahneman D. Tversky A. (1979). Prospect theory: an analysis of decision under risk. *Econometrica* 47 263–291 10.2307/1914185

[B18] Lane S. D. Yechiam E. Busemeyer J. R. (2006). Application of a computational decision model to examine acute drug effects on human risk taking. *Exp. Clin. Psychopharmacol.* 14 254–264 10.1037/1064-1297.14.2.254 16756429

[B19] Lin C.-H. Chiu Y.-C. Lee P.-L. Hsieh J.-C. (2007). Is deck b a disadvantageous deck in the Iowa Gambling Task? *Behav. Brain Funct*. 3 16 10.1186/1744-9081-3-16 PMC1839101 17362508

[B20] Luce R. D. (1959). *Individual Choice Behavior.* New York: Wiley.

[B21] Nelder J. A. Mead R. (1965). A simplex method for function minimization. *J. Comput.* 7 308–313 10.1093/comjnl/7.4.308

[B22] Rescorla R. A. Wagner A. R. (1972). “A theory of Pavlovian conditioning: variations in the effectiveness of reinforcement and nonreinforcement,” in *Classical Conditioning II: Current Research and Theory*, eds Black A. H. Prokasy W. F. (New York, NY: Appleton-Century-Crofts), 64–99.

[B23] Schwartz G. (1978). Estimating the dimension of a model. *Ann. Stat.* 6 461–464 10.1214/aos/1176344136

[B24] Steingroever H. Wetzels R. Horstmann A. Neumann J. Wagenmakers E.-J. (2013). Performance of healthy participants on the Iowa gambling task. *Psychol. Assess.* 25 180–193 10.1037/a0029929 22984804

[B25] Stout J. Busemeyer J. Lin A. Grant S. Bonson K. (2004). Cognitive modeling analysis of decision-making processes in cocaine abusers. *Psychon. Bull. Rev.* 11 742–747 10.3758/bf03196629 15581127

[B26] Stout J. C. Rodawalt W. C. Siemers E. R. (2001). Risky decision making in Huntington’s disease. *J. Int. Neuropsychol. Soc.* 7 92–101 10.1017/S1355617701711095 11253845

[B27] Toplak M. E. Jain U. Tannock R. (2005). Executive and motivational processes in adolescents with Attention-Deficit-Hyperactivity Disorder (ADHD). *Behav. Brain Funct.* 1:8 10.1186/1744-9081-1-8 PMC1183187 15982413

[B28] Upton D. J. Kerestes R. Stout J. C. (2012). Comparing the Iowa and Soochow gambling tasks in opiate users. *Front. Neurosci.* 6:34 10.3389/fnins.2012.00034 PMC3303110 22435045

[B29] Vassileva J. Gonzalez R. Bechara A. Martin E. M. (2007). Are all drug addicts impulsive? effects of antisociality and extent of multidrug use on cognitive and motor impulsivity. *Addict. Behav.* 32 3071–3076 10.1016/j.addbeh.2007.04.017 17507173 PMC2128047

[B30] Worthy D. A. Hawthorne M. J. Otto A. R. (2013a). Heterogeneity of strategy use in the Iowa gambling task: a comparison of win-stay/lose-shift and reinforcement learning models. *Psychon. Bull. Rev.* 20 364–371 10.3758/s13423-012-0324-9 23065763

[B31] Worthy D. A. Pang W. Byrne K. A. (2013b). Decomposing the roles of perseveration and expected value representation in models of the Iowa gambling task. *Front. Psychol.* 4:640 10.3389/fpsyg.2013.00640 PMC3786232 24137137

[B32] Worthy D. A. Maddox W. T. (2012). Age-based differences in strategy use in choice tasks. *Front. Neurosci.* 5:145 10.3389/fnins.2011.00145 PMC3252562 22232573

[B33] Yechiam E. Busemeyer J. R. (2008). Evaluating generalizability and parameter consistency in learning models. *Games Econ. Behav.* 63 370–394 10.1016/j.geb.2007.08.011

[B34] Yechiam E. Busemeyer J. R. Stout J. C. Bechara A. (2005). Using cognitive models to map relations between neuropsychological disorders and human decision-making deficits. *Psychol. Sci.* 16 973–978 10.1111/j.1467-9280.2005.01646.x 16313662

